# Linking Diabetic Retinopathy Severity to Coronary Artery Disease Risk Factors in Type 2 Diabetic Patients

**DOI:** 10.7759/cureus.65018

**Published:** 2024-07-21

**Authors:** Varun Bhaskar Lingineni, Sangram Mangudkar, Vijayashree S Gokhale, Satbir Malik, Ponvijaya Yadav

**Affiliations:** 1 General Medicine, Dr. D. Y. Patil Medical College, Hospital and Research Centre, Pune, IND

**Keywords:** non-proliferative diabetic retinopathy, coronary artery disease (cad), diabetes type 2, opthalmology ocular pathology, general internal medicine, risk factors for diabetic retinopathy

## Abstract

Background: Diabetes mellitus (DM) is a common metabolic disorder characterized by hyperglycemia, leading to chronic complications, notably cardiovascular diseases such as coronary artery disease (CAD). Diabetic retinopathy (DR), a leading cause of blindness, may serve as a non-invasive marker for CAD. This study investigates the correlation between DR and CAD to explore its diagnostic potential in diabetic populations.

Methods: A cross-sectional study was conducted over one year in a general hospital, involving 100 type 2 DM patients with retinopathy. DR was classified as mild non-proliferative diabetic retinopathy (NPDR), moderate NPDR, severe NPDR, or proliferative retinopathy, based on fundus examinations. Data on age, duration of diabetes, cholesterol levels, glycated hemoglobin (HbA1C), and ECG (electrocardiography) findings were collected. Statistical analysis included frequency analysis, chi-square tests for association between categorical variables, and significance testing with p-values. Data were analyzed using IBM SPSS Statistics for Windows, Version 20.0 (Released 2011; IBM Corp., Armonk, New York, United States). Descriptive statistics were characterized by categorical and continuous variables. The chi-square test determined associations between qualitative variables, with significance set at p<0.05.

Results: The mean age of patients was 57.13 ± 8.51 years. Age and duration of diabetes were significant predictors of retinopathy severity (p<0.001). Proliferative retinopathy was found exclusively in patients over 70 years. Lower cholesterol levels (<200 mg/dL) were significantly associated with less severe retinopathy (p=0.033), whereas higher cholesterol levels (>200 mg/dL) did not show a statistically significant association with retinopathy severity (p=0.772). Patients with HbA1C levels between 6.5% and 8.5% predominantly had milder forms of retinopathy, as indicated by the significant p-value (<0.001). In contrast, patients with HbA1C levels above 8.5% are more likely to have severe NPDR or proliferative diabetic retinopathy (PDR), but this association was not statistically significant (p=0.582). ECG abnormalities increased with retinopathy severity (p=0.002). Hypertension was significantly linked to cardiac changes in retinopathy patients (p<0.001), while smoking and family history of CAD were not significant factors. This study's cross-sectional design limits causality inference. The single-center sample of 100 patients may not be broadly generalizable. Reliance on self-reported data introduces potential recall bias, and confounding factors such as diet, physical activity, and additional comorbidities were not accounted for. The lack of a control group further limits comparative analysis. Future longitudinal studies with larger, diverse samples are needed.

Conclusion: Retinopathy in DM patients is significantly associated with cardiac changes and other risk factors such as hypertension, dyslipidemia, and poor glycemic control. Aggressive management of these factors is essential. Retinopathy can serve as a predictor of CAD in diabetic patients.

## Introduction

Diabetes mellitus (DM) comprises a group of common metabolic disorders characterized by chronic hyperglycemia. Chronic complications of DM affect many organ systems, with cardiovascular disease being the leading cause of morbidity and ischemic heart disease the most common cause of mortality [1]. The presence of DM significantly increases the risk of developing coronary artery disease (CAD) and is associated with higher risks of acute coronary syndrome and mortality following acute myocardial infarction (AMI) [2]. Therefore, individuals with DM benefit more from proven therapies for acute CAD, such as statins, antiplatelet agents, and aggressive glycemic control, which can help manage conditions including acute myocardial infarction and unstable angina [3,4].

DM is also the leading cause of blindness among individuals aged 20-74 years in the United States, with affected individuals being 25 times more likely to become legally blind compared to non-diabetic individuals [5]. Recent data indicates that the global prevalence of diabetic retinopathy (DR) among people with diabetes is approximately 35%, with vision-threatening DR affecting nearly 12% of this population [6]. In India, DM is the sixth leading cause of blindness, with the diabetic population projected to rise from 50.8 million in 2010 to 87.0 million by 2030 [7]. Additionally, recent estimates suggest that globally, 537 million adults were living with diabetes in 2021, projected to reach 783 million by 2045 [8]. The increasing prevalence of DM and DR highlights the urgent need for effective screening and management strategies.

There is continued interest in identifying methods and markers for ischemic heart disease to enhance patient risk stratification. Retinal vasculature changes, as seen in diabetic retinopathy (DR), have been proposed as an easily and safely measured surrogate for coronary circulation [9]. Previous studies have examined the association between DR and CAD, but the results have been conflicting. Some studies found a significant association [10,11], while others found no correlation [12], possibly due to shared risk factors for both DR and CAD.

Incorporating comprehensive CAD risk factors, utilizing a robust sample size, and conducting multivariate regression analysis are novel approaches to investigating the relationship between the severity of DR and predicting CAD in patients with type 2 DM (T2DM). Studies have shown the significance of various factors such as radiomic analysis of intermuscular adipose tissue (IMAT) [13], coronary computed tomographic angiography (CCTA) findings [14], computed tomography (CT)-based imaging parameters and radiomic features of pericoronary adipose tissue (PCAT) [15], and metabolic biomarkers combined with clinical characteristics [16] in assessing CAD risk. These approaches provide a comprehensive understanding of the interplay between DR severity and CAD prediction, offering valuable insights for non-invasive risk assessment in T2DM patients.

Given these mixed findings, the novelty of our study lies in addressing gaps by incorporating comprehensive CAD risk factors, a robust sample size, and multivariate regression analyses. Unlike previous studies, our methodology includes detailed stratification of DR severity and a diverse patient population from a single-center cohort, ensuring comprehensive clinical and biochemical evaluations. This approach allows for a more precise assessment of the independent effects of each risk factor on the relationship between DR and CAD. This study aims to investigate whether the severity of DR serves as a non-invasive marker for predicting CAD in patients with T2DM, potentially improving risk stratification and management of cardiovascular complications in diabetic patients.

## Materials and methods

This was a cross-sectional cohort study conducted over a period of one year from November 20, 2022, to November 20, 2023, at Dr. D. Y. Patil Medical College, Hospital and Research Centre, Pune, Maharashtra, India. The study involved 100 patients with T2DM who had been diagnosed with DR. The study was approved by the Institutional Ethics Sub-Committee of Dr. D. Y. Patil Medical College, Pune, India (approval number IESC/PGS/2022/21). Participants were thoroughly informed about the nature and purpose of the study, and written informed consent was obtained from each participant. Measures were taken to ensure confidentiality and privacy of patient information, and data were anonymized to maintain participant confidentiality.

Inclusion and exclusion criteria

Inclusion Criteria

Inclusion criteria were patients with T2DM diagnosed according to the American Diabetes Association (ADA) criteria. This included a random plasma glucose level exceeding 200 mg/dL (11.1 mmol/L) in patients with classic hyperglycemia symptoms or hyperglycemic crisis, a two-hour plasma glucose level over 200 mg/dL (11.1 mmol/L) during an oral glucose tolerance test (OGTT), a fasting plasma glucose level greater than 126 mg/dL (7.0 mmol/L) with fasting defined as no caloric intake for at least eight hours, and an HbA1C level exceeding 6.5%. Additionally, patients with confirmed DR (mild NPDR, moderate NPDR, severe NPDR, or PDR) were included in the study.

Exclusion Criteria

Exclusion criteria were established to refine the patient cohort further. Patients with type 1 DM were excluded, as well as those with uncontrolled hypertension, defined as blood pressure that cannot be reduced to the target level despite optimal treatment with a three-drug regimen including a diuretic in compliant patients. Additionally, patients with isolated systolic hypertension, where the target systolic blood pressure should be below 140 mmHg, were also excluded from the study.

Sample size

Based on the study conducted by Wong TY et al. [7], the sample size was calculated based on the prevalence of DR among patients with T2DM, estimated at 35%. Assuming a margin of error of 10% and a 95% confidence interval (CI), the minimum sample size required was calculated to be 87 patients. To ensure robustness, we increased this to 100 patients. This calculation was performed using WinPepi software, version 11.38 (J.H. Abramson, Brixton Health, United Kingdom). 

Data collection

The study sample consisted of 100 participants with T2DM who had varying severities of DR. The ophthalmologist-confirmed diagnosis of DR was based on the eye with the most severe manifestations. DR was classified into mild NPDR, moderate NPDR, severe NPDR, and PDR using fundus examinations conducted after mydriasis with tropicamide.

Detailed clinical histories were obtained through patient interviews and a review of medical records. The interviews included questions about the duration of diabetes, IHD, retinopathy, family history of T2DM and CAD, and smoking history. To minimize recall bias, patients were encouraged to refer to their medical records and prescriptions during the interviews. It is acknowledged that self-reported data on smoking history and family history may introduce recall bias. Additionally, we accounted for other potential confounding factors such as age, sex, duration of diabetes, and comorbid conditions. A comprehensive clinical examination was conducted for each participant, including measurements of body mass index (BMI), blood pressure, and a fundus examination. The evaluation also included signs of congestive cardiac failure (CCF), such as pedal edema, raised jugular venous pressure, and basal crepitations on auscultation.

Baseline diabetes-specific and acute coronary syndrome-related biochemical investigations were performed, including blood sugar levels (BSL) on admission, HbA1C, urine for albumin, renal function tests, and lipid profiles. The BSL was measured using the glucose oxidation method, HbA1C using high-performance liquid chromatography (HPLC), and lipid profiles using the CHOD-PAP method in a fasting state.

Cardiac assessments included a 12-lead ECG analyzed for rate, rhythm, axis, P wave, PR interval, QRS changes, ST segment, and T wave abnormalities. Abnormalities were defined as follows: T wave inversion of ≥1 mm in depth in two or more contiguous leads, QRS duration >120 ms, ST-segment elevation or depression of ≥1 mm in two or more contiguous leads, PR interval >200 ms, and QT interval corrected for heart rate (QTc) >440 ms in men and >460 ms in women. Additionally, two-dimensional (2D) echocardiography was performed to assess diastolic left ventricular function, ejection fraction, and the presence of left ventricular hypertrophy (LVH).

Statistical analysis

Data were entered into a Microsoft Excel 2010 spreadsheet (Microsoft Corporation, Redmond, Washington, United States) and imported into IBM SPSS Statistics for Windows, Version 20.0 (Released 2011; IBM Corp., Armonk, New York, United States) for analysis. Descriptive statistics including frequency analysis, percentage analysis, and mean analysis were used to characterize categorical and continuous variables. The chi-square test was employed to ascertain the association between qualitative variables, with significance set at p<0.05. 

Missing data were handled using multiple imputations; mean values were used for continuous variables, and the most frequent category for categorical variables. Sensitivity analyses assessed the impact of imputed data. To address multiple comparisons, the Bonferroni correction was applied by dividing the standard significance level (0.05) by the number of comparisons, reducing the likelihood of Type I errors.

## Results

Demographic and clinical characteristics

The study involved 100 patients with T2DM and DR. The mean age of patients was 57.13 ± 8.51 years. A significant proportion (50%, N=50) were aged 51-60, and 66 were male while 34 were female. The mean duration of diabetes was 12.5 years.

Age and retinopathy severity

Table 1 presents the distribution of patients by age and retinopathy severity. Patients aged 41-50 years had only mild NPDR, while those over 70 years exclusively had PDR. The data show that the severity of DR increases with age. There is a statistically significant association between age and retinopathy severity for the 41-50, 61-70, and 71-80 age groups, but not for the 51-60 age group. This highlights the need for regular and thorough eye exams for older diabetic patients, as the risk of severe retinopathy rises significantly with age. It is recommended that diabetic patients undergo comprehensive dilated eye examinations at least once a year. A thorough eye exam includes not only visual acuity testing and intraocular pressure measurement but also detailed retinal examination through dilated pupils, which allows for a better assessment of the retina, optic nerve, and macula.

**Table 1:** Distribution of patients by age and retinopathy severity The p-values indicate the significance of the association between age group and the severity of diabetic retinopathy (DR). A p-value of less than 0.05 is considered statistically significant. The distribution of diabetic retinopathy (DR) severity varies significantly across different age groups. Patients aged 41-50 years had only mild NPDR, with a highly significant association (p<0.001). In the age group of 51-60 years, DR severity was more varied, with no statistically significant association (p=0.823). In contrast, patients aged 61-70 and 71-80 showed a significant increase in severe NPDR and PDR, with p-values of 0.037 and 0.001, respectively, indicating that older patients are at a higher risk for severe retinopathy. NPDR: non-proliferative diabetic retinopathy; PDR: proliferative diabetic retinopathy

Age Group (years)	Mild NPDR, n (%)	Moderate NPDR, n (%)	Severe NPDR, n (%)	PDR, n (%)	Total (N)	p-value
41-50	18 (100%)	0 (0%)	0 (0%)	0 (0%)	18	<0.001
51-60	12 (24%)	20 (40%)	12 (24%)	6 (12%)	50	0.823
61-70	0 (0%)	0 (0%)	8 (36.36%)	14 (63.64%)	22	0.037
71-80	0 (0%)	0 (0%)	0 (0%)	10 (100%)	10	0.001
Total	30	20	20	30	100	-

**Figure 1:**
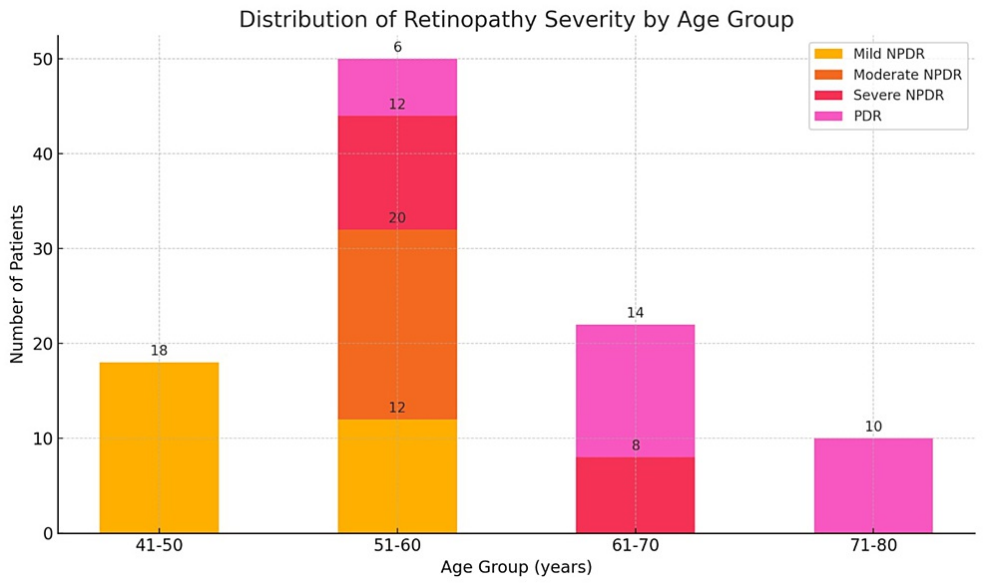
Stacked bar chart representing the distribution of diabetic retinopathy severity across different age groups In the 41-50 age group, all 18 patients (100%) had mild NPDR, with no instances of more severe retinopathy. The 51-60 age group exhibited a higher diversity in retinopathy severity, with 12 patients (24%) having mild NPDR, 20 patients (40%) moderate NPDR, 12 patients (24%) severe NPDR, and six patients (12%) PDR. For patients aged 61-70, severity increased significantly, with 8 patients (36.36%) having severe NPDR, and 14 patients (63.64%) having PDR, and no cases of mild or moderate NPDR. The 71-80 age group had the most severe cases, with all 10 patients (100%) having PDR and no instances of mild, moderate, or severe NPDR. NPDR: non-proliferative diabetic retinopathy; PDR: proliferative diabetic retinopathy

The stacked bar chart (Figure 1) represents the distribution of DR severity across different age groups among the study participants. The chart displays four categories of retinopathy severity: Mild NPDR, Moderate NPDR, Severe NPDR, and PDR. The chart clearly illustrates a correlation between increasing age and the severity of DR. Younger patients, specifically those aged 41-50 years, predominantly exhibit milder forms of retinopathy. In contrast, older patients, particularly those aged 71-80 years, predominantly suffer from more severe forms, especially PDR. The age group of 51-60 years shows a mix of severities, reflecting the progressive nature of the disease over time. Additionally, the age group of 61-70 years indicates a transition towards more severe forms of retinopathy, underscoring the critical importance of early detection and timely intervention.

Duration of diabetes and retinopathy severity

Table 2 shows the relationship between the duration of diabetes and retinopathy severity. The significant p-value(<0.001) for the ≤ 5 years category indicates a distinct connection between a shorter duration of diabetes and less severe retinopathy.

**Table 2:** Distribution of patients by duration of diabetes and retinopathy severity The p-value is considered significant at p<0.05; percentages have been calculated as per duration of DR category totals. The overall trend in the data shows that the severity of retinopathy increases with the duration of diabetes. However, the individual p-values for the 6-10 years and >10 years categories (0.643 and 0.783, respectively) are not statistically significant. This suggests that while there is a general trend, the specific associations within these duration categories may not be strong enough to be considered significant on their own. The significant p-value for the ≤ 5 years category indicates a clear relationship between shorter duration and milder retinopathy, but the longer durations do not show significant associations when analyzed separately. NPDR: non-proliferative diabetic retinopathy; PDR: proliferative diabetic retinopathy

Duration of DM (years)	Mild NPDR, n (%)	Moderate NPDR, n (%)	Severe NPDR, n (%)	PDR, n (%)	Total (N)	p-value
≤ 5	14 (87.5%)	0 (0%)	0 (0%)	2 (12.5%)	16	<0.001
6-10	16 (69.57%)	5 (21.74%)	0 (0%)	2 (8.70%)	23	0.643
> 10	0 (0%)	15 (24.59%)	20 (32.79%)	26 (42.62%)	61	0.783
Total	30	20	20	30	100	-

Cholesterol levels and retinopathy severity

The p-value of 0.033 indicates a significant association between lower cholesterol levels (<200 mg/dL) and retinopathy severity, while the p-value of 0.772 shows no significant association between higher cholesterol levels (>200 mg/dL) and retinopathy severity (Table 3).

**Table 3:** Distribution of patients by cholesterol levels and retinopathy severity The p-value is considered significant at p<0.05; percentages have been calculated as per cholesterol category totals Lower cholesterol levels (<200 mg/dL) were significantly associated with less severe retinopathy (p=0.033), whereas higher cholesterol levels (>200 mg/dL) did not show a statistically significant association with retinopathy severity (p=0.772). NPDR: non-proliferative diabetic retinopathy; PDR: proliferative diabetic retinopathy

Cholesterol (mg/dL)	Mild NPDR, n (%)	Moderate NPDR, n (%)	Severe NPDR, n (%)	PDR, n (%)	Total (N)	p-value
< 200	21 (33.33%)	17 (26.98%)	9 (14.29%)	16 (25.40%)	63	0.033
> 200	9 (24.32%)	3 (8.11%)	11 (29.73%)	14 (37.84%)	37	0.772
Total	30	20	20	30	100	-

Glycemic control and retinopathy severity

The results in Table 4 indicate that patients with HbA1C levels of 6.5-8.5% are significantly more likely to have less severe retinopathy (p<0.001), suggesting that maintaining HbA1C within this range can help reduce retinopathy severity in diabetic patients. Conversely, for patients with HbA1C levels above 8.5%, the lack of a significant association (p=0.582) implies that other factors may influence retinopathy severity, and focusing solely on HbA1C levels may not be adequate for managing the condition in this group.

**Table 4:** Distribution of patients by hbA1C levels and retinopathy severity The p-values indicate the significance of the association between HbA1C levels and the severity of diabetic retinopathy (DR). The p-value is considered significant at p<0.05 Patients with HbA1C levels between 6.5% and 8.5% predominantly have milder forms of retinopathy, as indicated by the significant p-value (<0.001). In contrast, patients with HbA1C levels above 8.5% are more likely to have severe NPDR or PDR, but the association is not statistically significant (p=0.582). NPDR: non-proliferative diabetic retinopathy; PDR: proliferative diabetic retinopathy

HbA1C (%)	Mild NPDR, n (%)	Moderate NPDR, n (%)	Severe NPDR, n (%)	PDR, n (%)	Total (N)	p-value
6.5-8.5	25 (52.08%)	9 (18.75%)	7 (14.58%)	7 (14.58%)	48	<0.001
> 8.5	5 (9.62%)	11 (21.15%)	13 (25%)	23 (44.23%)	52	0.582
Total	30	20	20	30	100	-

**Figure 2:**
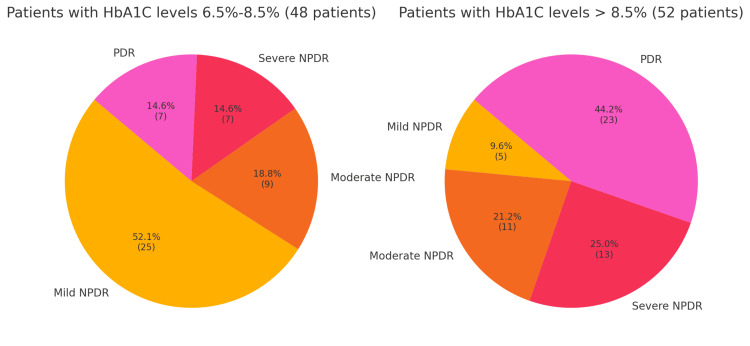
Pie charts representing correlation of HbA1c with severity of retinopathy Mild NPDR: minimal retinal changes; Moderate NPDR: More pronounced changes than mild NPDR but no immediate risk of vision loss; Severe NPDR: Significant retinal changes with a higher risk of progressing to PDR; PDR: Characterized by the growth of new blood vessels on the retina, which can lead to severe vision loss. NPDR: non-proliferative diabetic retinopathy; PDR: proliferative diabetic retinopathy; HbA1c: glycated hemoglobin

The pie charts shown in Figure 2 represent the proportion of patients with different severities of DR within two groups based on their HbA1C levels: 6.5-8.5% and greater than 8.5%. The goal is to illustrate how glycemic control influences the severity of retinopathy.

In the group of patients with HbA1C levels between 6.5% and 8.5%, 25 patients (52.1%) had mild NPDR, nine (18.8%) had moderate NPDR, seven (14.6%) had severe NPDR, and seven (14.6%) had PDR. This indicates that more than half of the patients in this group have mild NPDR, suggesting that better glycemic control is associated with less severe retinopathy, as only 29.2% of the patients have severe NPDR or PDR. Conversely, in the group of patients with HbA1C levels greater than 8.5%, five (9.6%) had mild NPDR, 11 (21.2%) had moderate NPDR, 13 (25.0%) had severe NPDR, and 23 (44.2%) had PDR. This shows that the majority of patients (69.2%) in this group have severe NPDR or PDR, highlighting a strong correlation between poor glycemic control and more severe forms of retinopathy.

Patients with HbA1C levels in the 6.5-8.5% range predominantly have milder forms of retinopathy. In contrast, patients with HbA1C levels above 8.5% show a significant increase in the severity of retinopathy, with a large proportion having severe NPDR or PDR. This data underscores the importance of maintaining good glycemic control to prevent the progression of diabetic retinopathy to more severe stages.

Cardiac changes and retinopathy severity

The results in Table 5 indicate a statistically significant association between mild NPDR and the presence of cardiac changes (p=0.002), suggesting a higher risk of cardiac changes even in the early stages of retinopathy. Conversely, the lack of significant associations in moderate NPDR (p=0.482), severe NPDR (p=0.372), and PDR (p=0.272) suggests that other factors may contribute to cardiac changes in more advanced retinopathy, highlighting the need for comprehensive cardiovascular assessment in these patients. Multivariate regression analysis indicated that retinopathy severity, hypertension, and elevated cholesterol levels were significant predictors of cardiac changes.

**Table 5:** Distribution of patients by retinopathy severity and cardiac changes The p-values indicate the significance of the association between the severity of retinopathy and the presence of cardiac changes. A p-value of less than 0.05 is considered statistically significant. For mild NPDR, the p-value is 0.002, indicating a statistically significant association with the presence of cardiac changes. In contrast, the p-values for moderate NPDR (0.482), severe NPDR (0.372), and PDR (0.272) indicate no statistically significant association between these severities of retinopathy and cardiac changes. NPDR: non-proliferative diabetic retinopathy; PDR: proliferative diabetic retinopathy

Retinopathy Severity	Cardiac Changes Present (N, %)	Cardiac Changes Absent (N, %)	Total (N)	p-value
Mild NPDR	7 (23.33%)	23 (76.67%)	30	0.002
Moderate NPDR	6 (30%)	14 (70%)	20	0.482
Severe NPDR	8 (40%)	12 (60%)	20	0.372
PDR	21 (70%)	9 (30%)	30	0.272
Total	42	58	100	-

ECG abnormalities

ECG abnormalities, including arrhythmias, left axis deviation (LAD), LVH, and ST-segment changes, were analyzed. The prevalence of ECG abnormalities increased with the severity of retinopathy. Table 6 provides a detailed distribution. Significant associations were found between the severity of retinopathy and both LAD and LVH. These findings highlight the importance of monitoring and managing cardiovascular complications in patients with advanced retinopathy. Other ECG changes did not show significant associations with retinopathy severity in this study.

**Table 6:** Distribution of ECG(electrocardiography) abnormalities by retinopathy severity The p-values indicate the significance of the association between each ECG change and the severity of retinopathy. A p value <0.05 is considered significant. The study found no statistically significant association between retinopathy severity and arrhythmia (p=0.260), abnormal QRS I (p=0.112), ST changes (p=0.824), abnormal T waves (p=0.188), and LBBB (p=0.300). However, there was a significant association between LAD (p=0.013) and LVH (p=0.003), indicating that the prevalence of LAD and LVH increases with the severity of retinopathy. NPDR: non-proliferative diabetic retinopathy; PDR: proliferative diabetic retinopathy; LBBB: left bundle branch block; LAD: left axis deviation; LVH: left ventricular hypertrophy

Retinopathy	Arrhythmia, n (%)	LAD, n (%)	LVH, n (%)	Abnormal QRS I, n (%)	ST Changes, n (%)	Abnormal T Waves, n (%)	LBBB, n (%)
Mild NPDR	0 (0%)	4 (13.33%)	2 (6.67%)	3 (10%)	4 (13.33%)	6 (20%)	1 (3.33%)
Moderate NPDR	0 (0%)	2 (10%)	2 (10%)	3 (15%)	5 (25%)	4 (20%)	0 (0%)
Severe NPDR	2 (10%)	4 (20%)	4 (20%)	0 (0%)	6 (30%)	7 (35%)	0 (0%)
PDR	2 (6.67%)	12 (40%)	12 (40%)	6 (20%)	7 (23.33%)	12 (40%)	2 (6.67%)
Total	4	22	20	12	22	29	3
p-value	0.260	0.013	0.003	0.112	0.824	0.188	0.300

Multivariate analysis

Multivariate logistic regression was used to assess the independent effects of retinopathy severity, duration of diabetes, HbA1C levels, and cholesterol levels on cardiac changes. The results indicated that severe retinopathy (OR=2.5, 95%CI: 1.2-5.1), duration of diabetes >10 years (OR=3.1, 95%CI: 1.5-6.3), and HbA1C >8.5% (OR=2.8, 95%CI: 1.4-5.5) were significant predictors of cardiac changes.

Age and duration of diabetes are significant predictors of retinopathy severity. Elevated cholesterol levels and poor glycemic control are strongly associated with severe retinopathy. Additionally, there is a significant association between retinopathy severity and cardiac changes, with ECG abnormalities increasing in prevalence as retinopathy severity worsens. Severe retinopathy, longer duration of diabetes, and poor glycemic control are significant predictors of cardiac changes. These findings underscore the importance of monitoring and managing cardiovascular risk factors in patients with diabetic retinopathy to mitigate cardiac complications.

Multicollinearity assessment

To ensure the robustness of our multivariate logistic regression analysis, we assessed potential multicollinearity among the predictors. Variance inflation factor (VIF) values were calculated for each predictor variable (retinopathy severity, duration of diabetes, HbA1C levels, and cholesterol levels). All VIF values were found to be below 5, indicating no significant multicollinearity among the predictors. This confirms the independence of each variable's contribution to the regression model, ensuring reliable estimation of the effects of retinopathy severity, duration of diabetes, HbA1C levels, and cholesterol levels on cardiac changes.

## Discussion

This cross-sectional study examined 100 patients with DR aged between 41 and 80 years, with a mean age of 57.13 ± 8.51 years. A significant proportion (50%, N=50) were aged 51-60, and 66 were male while 34 were female. The mean duration of diabetes was 12.5 years, with all patients over 70 having proliferative PDR. Age was found to be a significant risk factor for the severity of retinopathy (p<0.001), correlating with a longer duration of diabetes. In a study conducted by Bamashmus et al. [17], similar findings were reported, with the mean age of patients being 54.4 years and 52% of patients having diabetes for more than 10 years, with a mean duration of 9.9 years. The similarities in age and diabetes duration between the two studies may be due to the natural progression of diabetes and its complications, which tend to manifest more severely in older patients and those with a longer disease duration. However, slight differences in the mean age could be attributed to variations in the study populations, geographic locations, and healthcare access, which may influence the timing and severity of retinopathy diagnosis.

In the present study, the severity of retinopathy was significantly associated with the duration of diabetes, with 61 patients having diabetes for more than 10 years. Among these, 26 had PDR, 15 had moderate NPDR, and 20 had severe NPDR (p<0.001). Cholesterol levels greater than 200 mg/dL were found in 37 patients, and the proportion of these patients increased from mild NPDR (30%, N=9) to PDR (46.66%, N=14), showing a statistically significant association with retinopathy severity (p=0.033). This is consistent with the findings of the Hoorn study by van Leiden et al. [18], which also reported an association between retinopathy and serum cholesterol levels.

Poor glycemic control was evident in our study, with 52 patients having HbA1C levels greater than 8.5%. The association between DR and HbA1C levels was statistically significant (p<0.001), aligning with the results of a study by Henricsson et al. [19], which found that both the duration of diabetes and levels of glycosylated HbA1C were strongly related to retinopathy. The potential mechanisms linking poor glycemic control to severe DR are multifaceted. Chronic hyperglycemia leads to the formation of advanced glycation end-products, which contribute to vascular damage by increasing oxidative stress and inflammation. This can result in endothelial cell dysfunction, capillary occlusion, and retinal ischemia, which are precursors to neovascularization seen in PDR. Additionally, hyperglycemia activates various inflammatory pathways, with cytokines such as vascular endothelial growth factor (VEGF) playing a crucial role in DR progression [20].

Additionally, ECG changes were observed in 42 patients, with the percentage of patients having ECG changes increasing from 23.33% in mild NPDR to 70.0% in PDR. There was no significant gender difference in ECG changes, with 42.42% of males and 41.17% of females affected. The study by Mohammad et al. reported a significant correlation between severe retinopathy and cardiac autonomic neuropathy, suggesting that severe retinopathy can be an indicator of cardiac complications in diabetic patients [21].

Controlled hypertension was present in 29 patients, of whom 21 (72%) had cardiac changes, indicating a significant association between hypertension and retinopathy in relation to cardiac changes (p<0.001). This finding is supported by the UK Prospective Diabetes Study (UKPDS:23), which reported a significant association between CAD and hypertension, with estimated hazard ratios of 1.82 for systolic blood pressure [22].

In the present study, 29 patients reported a history of tobacco addiction, either through smoking or chewing, with 10 of these patients (34.48%) showing cardiac changes. Among the 71 non-smokers, 32 (45.07%) had cardiac changes, and the association between smoking and retinopathy in relation to cardiac changes was not statistically significant (p=0.33). This is consistent with the Chennai Urban Population Study (CUPS) by Viswanathan Mohan et al., which found no association between smoking and CAD [23]. The lack of significance may be due to a small sample size or unmeasured smoking intensity and duration. A family history of CAD also showed no significant correlation with retinopathy and cardiac changes (p=0.218), differing from Yarnell et al.'s findings [24], possibly due to genetic and environmental variations. 

The Hoorn study investigated the contribution of blood pressure, lipids, and obesity to retinopathy in diabetic and non-diabetic individuals [18]. It found that elevated blood pressure, BMI, cholesterol, and triglyceride levels are associated with retinopathy. The prevalence of retinopathy increased with worsening glucose tolerance and was highest in individuals with known DM. Retinopathy worse than minimal NPDR was associated with higher systolic blood pressure, BMI, and total cholesterol. This study aligns with our findings that elevated cholesterol levels and poor glycemic control are significantly associated with severe retinopathy. However, it also emphasizes the impact of blood pressure and obesity, which were not the primary focus of our study.

The 10-year study by Henricsson et al. examined the long-term progression of DR and its association with lipid levels [19]. Higher levels of low-density lipoprotein (LDL) cholesterol and triglycerides were associated with the progression of DR over the 10-year study period. Patients with higher baseline lipid levels were more likely to experience worsening of retinopathy. This study reinforces our findings on the significant association between high cholesterol levels and severe retinopathy. It supports the potential benefit of managing lipid levels in diabetic patients to prevent retinopathy progression.

Mohammad et al. studied the association between DR and cardiac autonomic neuropathy [21]. The study found a significant correlation between severe retinopathy and the presence of cardiac autonomic neuropathy, suggesting that severe retinopathy can be an indicator of cardiac complications in diabetic patients. This study supports our findings on the association between severe retinopathy and cardiac changes. Both studies highlight the need for comprehensive cardiovascular risk management in patients with advanced retinopathy.

Our study reveals a significant association between poor glycemic control and the severity of DR. Patients with higher HbA1C levels (>8.5%) were more likely to have severe forms of retinopathy, such as severe NPDR and PDR. In contrast, those with HbA1C levels between 6.5-8.5% predominantly exhibited milder forms of retinopathy. This finding underscores the critical role of maintaining optimal glycemic control in preventing the progression of DR. From a clinical perspective, these results emphasize the importance of stringent glycemic management in diabetic patients to mitigate the risk of developing severe retinopathy. Regular monitoring of HbA1C levels and early intervention in cases of poor glycemic control can be pivotal in preventing the advancement of retinopathy. Furthermore, the association between DR severity and cardiac changes suggests that patients with advanced retinopathy are at a heightened risk for cardiovascular complications, necessitating comprehensive cardiovascular risk management in these individuals. Older age and longer duration of diabetes were also significant predictors of retinopathy severity, highlighting the necessity for regular and comprehensive eye examinations in elderly diabetic patients and those with a long history of diabetes.

Future research should focus on longitudinal studies to track the progression of DR in relation to glycemic control and other risk factors. This will help establish a clearer temporal relationship between hyperglycemia and retinopathy severity. Interventional trials are also needed to assess the effectiveness of different glycemic control strategies in preventing or slowing DR progression. Exploring novel biomarkers for early detection and understanding the underlying mechanisms driving DR will aid in developing targeted therapies. Moreover, investigating the impact of lifestyle interventions, such as diet and physical activity, on DR progression could provide insights into non-pharmacological approaches for managing the condition.

Limitations

Our study's cross-sectional design limits the ability to infer causality between glycemic control and retinopathy severity. Longitudinal studies are necessary to establish temporal relationships. Additionally, the relatively small sample size and single-center setting may limit the generalizability of our findings. Larger, multicenter studies are required to confirm these results in diverse populations. The reliance on self-reported data for certain variables introduces potential recall bias, underscoring the need for objective measurements in future research. Furthermore, the study did not account for all potential confounding factors such as diet, physical activity, and other comorbid conditions, which should be controlled in future studies to isolate the effects of glycemic control on retinopathy severity. By addressing these limitations and focusing on the recommended areas for future research, we can gain a deeper understanding of the complex interplay between glycemic control and DR, ultimately leading to improved management strategies for patients with diabetes.

## Conclusions

The presence of retinopathy is significantly associated with cardiac changes in patients with DM. Our study found that patients with retinopathy also exhibit a high prevalence of other risk factors, including hypertension, dyslipidemia, and poor glycemic control. Therefore, in patients with retinopathy, it is crucial to aggressively search for and manage other cardiovascular risk factors to mitigate the risk of further complications. The cardiac changes observed in this study may be attributed to the long duration of diabetes, elevated cholesterol levels, and the presence of hypertension. While these changes can be linked to various cardiovascular risk factors, retinopathy itself emerges as a significant predictor of CAD in patients with DM. Consequently, regular cardiovascular assessments and comprehensive management strategies are essential for this patient population.
